# Supplementary figures and images for: Population structure, case clusters, and genetic lesions associated with Canadian Salmonella 4,[5],12:i:- isolates
Source: PLoS One. 2021 Apr 6;16(4):e0249079. doi: 10.1371/journal.pone.0249079 (PMC8049487; doi:10.1371/journal.pone.0249079)

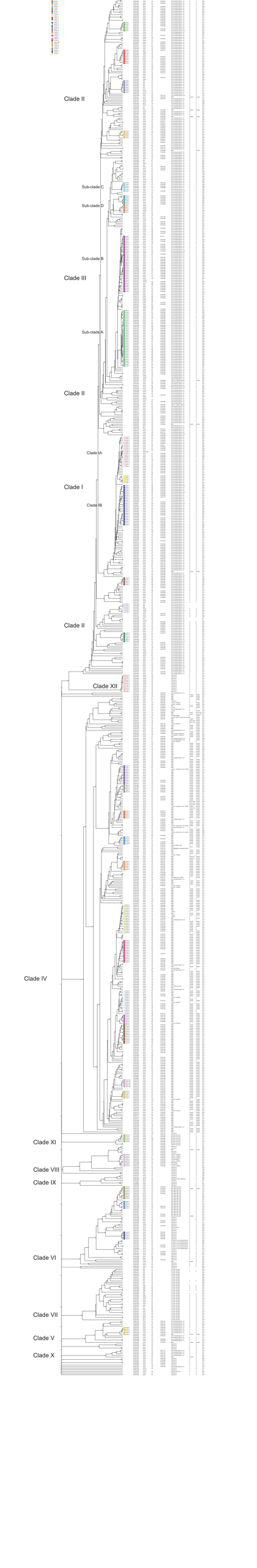

Supplement: S1 Fig — The dendrogram was created in BioNumerics v7.6.3 and annotated using Adobe Creative Cloud Illustrator CC. (PDF) [file pone.0249079.s001.pdf]

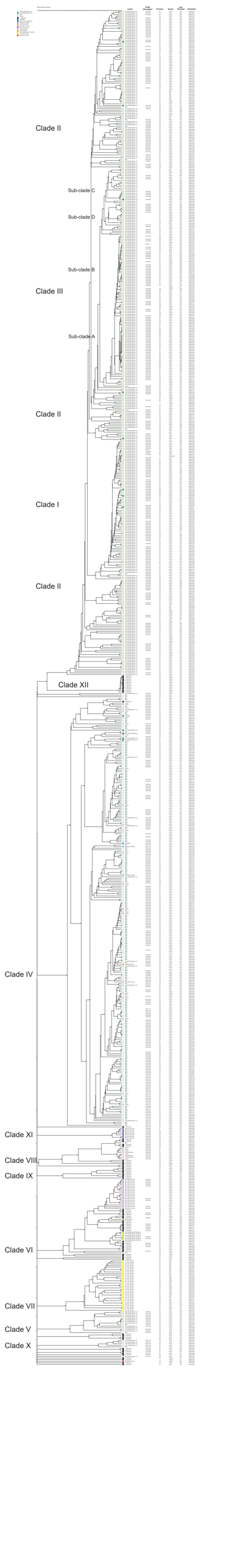

Supplement: S2 Fig — The dendrogram was created in BioNumerics v7.6.3 and annotated using Adobe Creative Cloud Illustrator CC. (PDF) [file pone.0249079.s002.pdf]

Short prophage insertion replacing *fljAB*

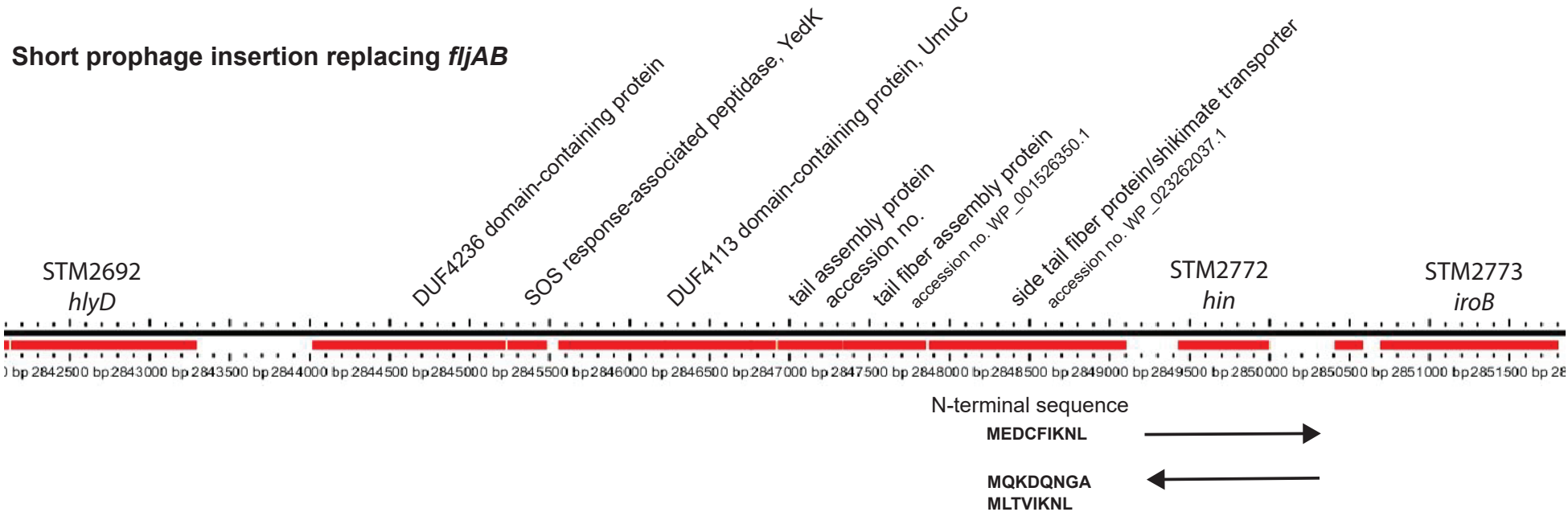

Supplement: S3 Fig — The figure was obtained using GView Server and annotated using Adobe Creative Cloud Illustrator CC. (PDF) [file pone.0249079.s003.pdf]
